# Uptake of cervical cancer screening service and associated factors among age-eligible women in Ethiopia: systematic review and meta-analysis

**DOI:** 10.1186/s13027-020-00334-3

**Published:** 2020-11-13

**Authors:** Asteray Assmie Ayenew, Biruk Ferede Zewdu, Azezu Asres Nigussie

**Affiliations:** 1 Midwifery department, College of Medicine and Health Sciences, Bahir Dar University, Bahir Dar, Ethiopia; 2 College of Medicine and Health Sciences, Department of Orthopedics, Bahir Dar University, Bahir Dar, Ethiopia

## Abstract

**Background:**

Cervical cancer is the leading cause of cancer deaths among women in developing countries. Since cervical cancer is a preventable disease, screening is an important control and prevention strategy, recommended by the World Health Organization (WHO) for all women aged 30 years and older, and even earlier for some high-risk women. Therefore the aim of this study was to assess the uptake of cervical cancer screening among age-eligible women in Ethiopia.

**Method:**

Review identification was performed through the search of online databases PubMed, Google Scholar, HINARI, EMBASE, Science Direct, Cochrane library, African Journals, and other gray and online repository accessed studies were searched using different search engines. For critical appraisal of studies, Newcastle-Ottawa Quality Assessment Scale (NOS) was used. The analysis was conducted by using STATA 11 software. To test the heterogeneity of studies, the Cochran Q test and I^2^ test statistics were used. To detect publication bias of the studies, the funnel plot and Egger’s test were used. The pooled prevalence of cervical cancer screening and the odds ratio (OR) with a 95% confidence interval were presented using forest plots.

**Result:**

Twenty-four studies with a total of 14,582 age-eligible women were included in this meta-analysis. The pooled national level of cervical cancer screening among age-eligible women in Ethiopia was 13.46% (95%CI:11.06,15.86). Knowledge on cervical cancer and screening (OR = 4.01,95%CI:2.76,5.92), history of multiple sexual partners (OR = 5.01, 95%CI:2.61,9.61), women’s age (OR = 4.58, 95%CI:2.81,7.46), history of sexually transmitted disease (OR = 4.83,95%CI:3.02,7.73), Perceived susceptibility to cervical cancer (OR = 3.59, 95%CI:1.99,6.48), getting advice from health care providers (OR = 4.58, 95%CI:3.26, 6.43), women’s educational level (OR = 6.68,95%CI:4.61,9.68), women’s attitude towards cervical cancer and screening (OR = 3.42, 95%CI:2.88,4.06) were the determinant factors of cervical cancer screening uptake among age-eligible women in Ethiopia.

**Conclusion:**

The pooled prevalence of cervical cancer screening was remarkably low among age-eligible women in Ethiopia. Thus, to increase the uptake of cervical cancer screening among age-eligible women regularly, it is better to create awareness programs for early detection and treatment of cervical cancer, and educational interventions that teach the step-by-step practice of cervical screening to increase women’s attitude for screening. Additionally, it is better to inform every woman is susceptible to cervical cancer, especially after starting sexual intercourse, and screening remains fundamental in the fight against cervical cancer before becoming invasive. Moreover, counseling and improving the confidence of women by health care providers to undergo screening is recommended.

## Background

Worldwide, cervical cancer is among the most common cancers and disproportionately affects the African women. As Africa is experiencing an epidemiologic transition, with aging populations that are susceptible to lifestyle diseases, and the continent accounts for an increasing proportion of global cancer cases and deaths [1]. Cervical cancer was the second prevalent and the leading cause of cancer deaths in Africa in 2018 [2]. By 2025, it is estimated that, about 78,879 women living in Africa will be diagnosed with cervical cancer annually, and 61,671 will die of cancer of cervix [3]. Regional variations in cervical cancer are especially marked; Sub-Saharan Africa (a region where Ethiopia is located) has the highest rates of cervical cancer in the world and cervical cancer is the number one cancer-related cause of mortality in the region [4].

In Africa, the epidemic of cervical cancer is both profound and complex, as a disease with both infectious and non-infectious etiologic components and risk factors. The African cervical cancer epidemic is characterized by the double burden of communicable and non-communicable disease [5], preventive health service delivery challenges [6–8], human resource for health shortages [9], lack of access to treatment, and low cervical cancer awareness among the population and health providers [10, 11].

In Ethiopia, the incidence and mortality from cervical cancer is 26.4 and 18.4/100,000 respectively. These figures are probably lower than the actual number of cases, given the low level of awareness, limited access to screening services, coast, and lack of a national cancer registry [12, 13]. According to the only oncology centre in the country (the Tikur Ambessa (Black Lion) Specialized Hospital), about 80% of reported cases of cancer are diagnosed at advanced stages, when very little/nothing can be done to treat the disease. This is largely due to the inadequate screening and early detection and treatment services, low awareness of cancer signs and symptoms, inadequate diagnostic facilities and poorly structured referral system [14].

Human Papillomavirus (HPV) infection, a common and often asymptomatic sexually transmitted infection is the most cervical cancer cause. Most sexually active men and women will be infected at a point in their lifetime and some may be repeatedly infected. For both women and men the peak time for acquiring infection is shortly after becoming sexually active. Human Papillomavirus (HPV) infection is responsible for 99% of cervical cancer and accounts for approximately half of the infection-related burden of cancer in women. There are over 100 types of HPV. According to their association with genital tract cancer, the genital-type HPVs are divided into high, intermediate, and low-risk types. High risk types of HPV (HPV-16, − 18, − 31, − 45) account for more than 90% of cervical carcinoma [15]. Human Papillomavirus (HPV) 16/18 prevalence among Ethiopian women has been estimated at 45.3%, highlighting the importance of secondary prevention in this population [16].

Before progression to invasive disease, cervical cancer has a long preinvasive phase, enabling detection of precancerous changes by screening. Screening is an important control and prevention strategy, recommended by the World Health Organization (WHO) for age of 30 years and above women, and beginning even earlier for some high-risk women such as women living with HIV, or with a history of early sexual intercourse [17]. While screening by cytology (‘Pap smear’) has prevented up to 80% of cervical cancers in high-resource settings, this approach is not currently feasible in Africa including Ethiopia due to the lack of trained personnel and inadequate infrastructure [4]. Moreover, the low sensitivity of cytology necessitates regular (2–3 yearly) screening intervals, which is problematic in Ethiopia because of poor follow-up, poor awareness and limited recall systems [18]. The “Screen-and-treat” approaches using either HPV testing or visual inspection with acetic acid (VIA) followed by precancerous lesions are a cost-effective prevention strategy in low-resource settings [7, 8, 19].

Ethiopia, being a developing country, has adopted cheaper but effective techniques for screening of cervical cancer screening called Visual Inspection with Acetate (VIA), with the aim of employing routine screening for early detection of asymptomatic women and on-the-spot treatment of cervical pre-cancerous lesions.

Guidelines for cervical cancer screening (CCS) in Ethiopia advocate a ‘screen-and-treat’ approach where women aged 30 to 49 years are screened using VIA and treated with cryotherapy. The guidelines recommend annual screening for HIV-positive women and 3-yearly for all other women, but in actuality, screening is erratic and frequently determined by the availability of resources. Therefore, this systematic review and meta-analysis aimed to estimate the pooled prevalence of cervical cancer screening uptake and to identify the determinant factors in Ethiopia.

## Methods

### Search strategy

International Online databases (Pub Med, EMBASE, Science Direct Cochrane library, HINARI, Google Scholar, and African Journals) were used to search articles on cervical cancer screening uptake. Searching terms were based on adapted PICO questions to search through the aforementioned databases to accesses all-important articles. For the online database search the keywords; “cervical cancer screening”, “prevalence”, “uptake”, “practice”, “VIA”, “cervical cancer”, “pre cervical cancer screening”, “and 15–49 years old women”, “barriers”, “knowledge”, “attitude”, “determinants”, “associated factors and Ethiopia”. Additionally, we used “AND” or “OR” Boolean operators.

### Inclusion and exclusion criteria

Both case-control and cross-sectional studies were incorporated. Studies reported the prevalence and/or associated factors, or determinant factors of cervical cancer screening uptake in Ethiopia were included in this study. Only English language research articles and literature were included. Whereas duplicated studies, anonymous reports, articles without full text, and abstract, and editorial reports were excluded from the study.

### Data extraction and quality assessment

After collecting findings from all the databases, the articles were exported to a Microsoft Excel spreadsheet. Two independent reviewers (AAA & AAN) extracted the data and reviewed all the screened and included articles. Disagreements between reviewers were handled by the third reviewer (BFZ). Finally, a consensus was reached through discussion between the authors. Newcastle-Ottawa Quality Assessment Scale (NOS) for observational studies was used to assess the methodological quality of a study and to determine the extent to which a study addressed the possibility of bias in the design, conduct, and analysis. Three authors independently assessed the articles for inclusion in the review. Articles that scored seven and more (NOS) quality assessments were considered as a good study and low risk and included in this study.

### Outcome of measurement

The measurement outcome of this systematic review and meta-analysis had two main outcome variables. Cervical cancer screening uptake was the first outcome of the study, whereas associated factors of cervical cancer screening uptake were the second outcome of the study. For common factors, the odds ratio was calculated from the reported studies. The outcome of this study was to focus on single studies estimating the prevalence of cervical cancer screening uptake among age-eligible women in Ethiopia.

### Publication bias and heterogeneity

To assess the heterogeneity of the study, the Cochrane Q test, and I2 with its corresponding *p*-value were used. A value of 25, 50, and 75% was used to declare the heterogeneity test as low, medium, and high heterogeneity, respectively. To assess the existence of publication bias, funnel plot and Egger regression asymmetry tests were employed. Moreover, with the evidence of heterogeneity, the random effect model analysis was computed.

### Data analysis

The data were entered using a Microsoft Excel spreadsheet. For data analysis, we used Stata 11 software. The estimated prevalence of each study was presented using forest plots with a 95% confidence interval (CI). Additionally, subgroup analysis was computed using the year of study and the study region, with the evidence of heterogeneity.

## Result

### Characteristics of the included studies

**Fig. 1:**
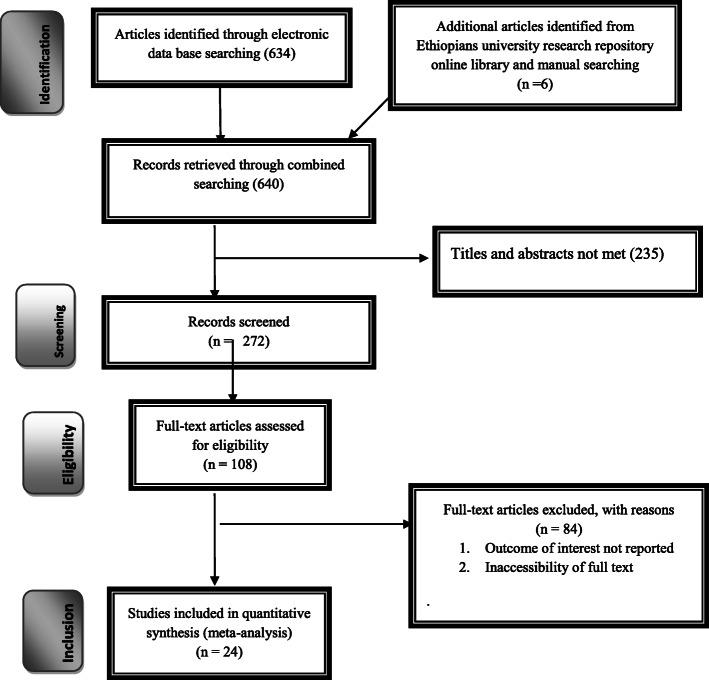
PRISMA Flow diagram for identification and selection of articles included in this review

Six hundred forty studies were retrieved at Pub Med, EMBASE, Science Direct, Cochrane library, HINARI, Google Scholar, African Journals, and other gray and online repositories accessed articles regarding the prevalence and determinant factors of cervical cancer screening in Ethiopia. After duplicates were expunged, 507 studies remained. After review of their abstracts and titles, 235 articles were excluded. Therefore, 108 full-text articles were assessed and for inclusion criteria, which resulted in the further exclusion of 84 articles primarily due to two reasons reported in (Fig. 1). As a result, 24 studies were met the inclusion criteria to undergo the final systematic review and meta-analysis. Among the included studies articles, two were case-control and 20 were cross-sectional study design. Studies were conducted from different regions of Ethiopia (Amhara, Tigray, SNNPR (South Nation Nationalities, people, and representatives), Oromia, and Addis Ababa). Overall, this review included a total of 14, 582 age-eligible women in Ethiopia (Table 1).

**Table 1:** Study characteristics included in the systematic review and meta-analysis

Author (year of study)	Region	Study area	Study design	Sample size	Prevalence(p)%	Study participants
Friederike R. et al.(2020) [20]	Oromia	butajira	Community cross-sectional	354	4	Women in rural Ethiopia age 30–49
Tekle T.et al.(2019) [21]	SNNP	Wolaita Zone	Facility based cross-sectional	520	22.9	30–49 age women
Abyu.A et al.(2019) [22]	SNNP	Hawassa hospital	Intituational based cross-sectional	350	40.1	HIV positive womenattending ART cliniv
Jeylan K. et al.(2020) [23]	SNNP	Shabadino District	Community based cross-sectional	536	10.5	30–40 age women
Belete A. et al.(2019) [24]	Amhara	Bahir Dar	Case control	230	N/A	Commercial sex workers
Daniel A.et al.(2017) [25]	Amhara	Gondar	Institutional based cross-sectional	302	23.5	HIV positive women
Kalkidan S.et al.(2019) [26]	Oromia	bishoftu	Institutional based cross-sectional	475	25	HIV positive women attending adult anti retroviral treatment clinic
Fasika T.et al.(2019) [27]	Amhara	dessie	Community based cross-sectional	620	11	15–49 age women
Meried E.et al.(2017) [28]	Amhara	Dabat District	Institutional based cross-sectional	660	12.1	30–49 age women
Tadesse N.et al.(2019) [29]	Oromia	Jimma	Community based cross-sectional	746	15.5	30–49 years of age women
Hinsermu B.et al.(2016) [30]	Tigray	mekele	Community based cross-sectional	1286	19.8	age eligible women
Bewket Y.et al.(2020) [31]	Amhara	Debre-Markos	Community based cross-sectional	822	5.4	30–49 age women
Tinsae S.et al.(2018) [32]	SNNP	Arbaminich,Gamo Gofa	Facility based cross-sectional	364	9.6	Female health care providers
Alehegni B.et al920180 [33]	Amahra	Finote-Selam	Community based cross-sectional	1152	8	30–49 age women
Simachew A.et al.(2019) [34]	Amahra	Debre-Markos	Community based cross-sectional	517	21.2	Reproductive age women
Abebe B. Et al(2020) [35]	Addis Ababa	Pawulos hospital	hospital-based cross sectional study	425	12.2	18–49 age women
Zeleke G.et al. (2016) [36]	SSNNP	Arba Minchi	Community based cross-sectional	660	7.9	Married reproductive age women
Netsanet B.et al.(2015) [37]	Addis Ababa	Tikur-Ambessa	hospital-based cross sectional study	333	24.8	HIV positive women
Abebe D. et al.(2016) [38]	Amhara	Gondar hospital	hospital-based cross sectional study	425	10	HIV positive women
Tsidkayehu B. et al.(2019) [39]	Addis Ababa	Addis Ababa	Institutional based cross-sectional	301	9.3	Health extension worker
Mohammed D.et al. (2018) [40]	Oromia	Butajira	Community based cross sectional	821	15.1	Women living in Butajira towen
Hirut T.et al.(2019) [41]	Tigray	Tigray public health hospita	Case control	624	N/A	Women attending public health hospital
Dawit G.et al. (2019) [42]	Tigray	Axum university	Institutional based cross-sectional	344	17.2	Health science students
Gizachew A.et al. (2019) [43]	Oromia	Ambo public hospital	hospital-based cross sectional study	423	5.1	women-living-with-hiv-in-public-hospitaa

### Meta-analysis

#### Publication bias

**Fig. 2:**
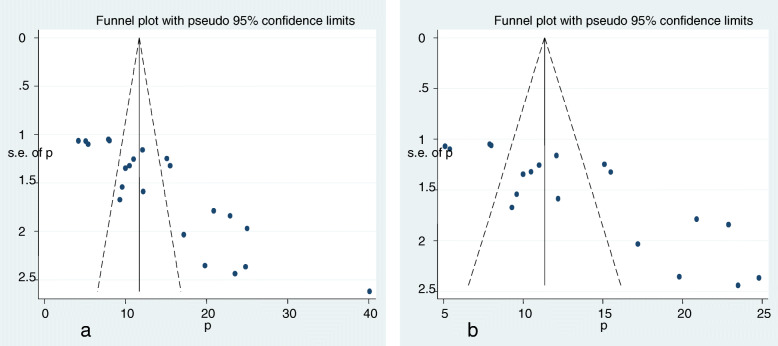
Funnel plot before adjustment (**a**) and after adjustment (**b**) for publication bias

Primarily, among 24 studies, two case-control studies [24, 41] were not considered in the prevalence estimation. Additionally, three studies [20, 22, 26] were excluded from prevalence estimation after checking funnel plot and the significance of Egger’s regression test. But, they were not excluded from meta-analysis for risk factors. Significant publication bias with an Egger’s regression *p*-value < 0.001 was seen when all studies were considered (Fig. 2a). After adjustment, Egger’s regression p-value was 0.15, indicated a reduced publication bias (Fig. 2b).

#### Prevalence of regular cervical cancer screening uptake in Ethiopia

**Fig. 3:**
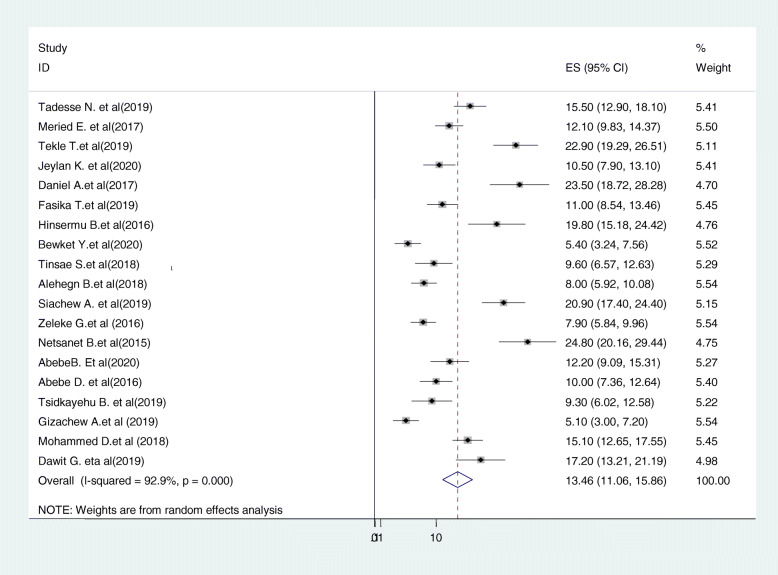
Forest plot displaying the pooled prevalence of cervical cancer screening uptake in Ethiopia

Consequently, 19 studies [21, 23, 25, 27–39, 42–44] were included in the final meta-analysis. A wide-ranging prevalence of cervical cancer screening uptake was observed across different studies included in this review. The pooled prevalence of cervical cancer screening uptake in Ethiopia was 13.46% (95%CI: 11.06, 15.86, I^2^ = 92.9%, p < =0.001) using a random effect model (Fig. 3).

#### Subgroup analysis

**Fig. 4:**
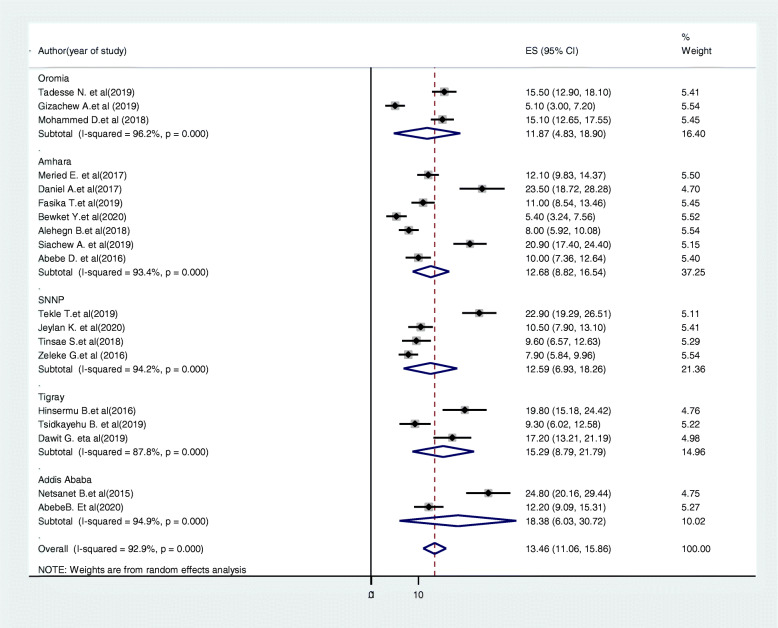
Forest plot of the subgroup analysis based on the region study

**Fig. 5:**
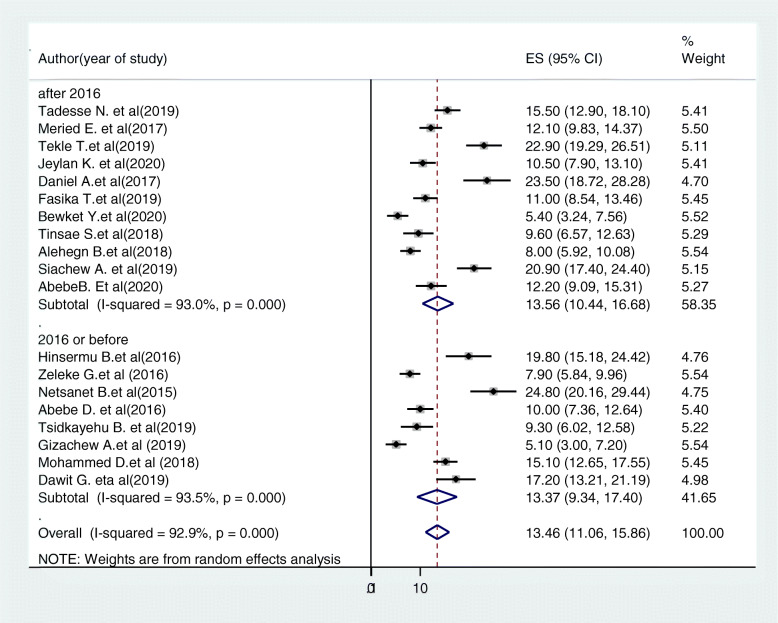
Forest plot of the subgroup analysis based on the year of study

Subgroup analysis was conducted based on the study region and year of study. Accordingly, the highest cervical cancer screening uptake was in Addis Ababa 18.38% (95%CI:6.03,30.72, I^2^ = 94.9, P < =0.001) and the lowest was in Oromia region 11.87% (95%CI: 4.83,18.9, I^2^ = 96.2%, p < = 0.001) (Fig. 4). Based on year of study the pooled prevalence of cervical cancer screening uptake in studies conducted before 2016 was 13.37% (95%CI:9.34,17.4, I2 = 93.5%, P < =0.001) and the prevalence was 13.57% (95%CI:10.44,16.68, I^2^ = 93, P < =0.001) in studies after 2016 (Fig. 5).

### Determinants of cervical cancer screening uptake

#### Relationship between knowledge on cervical cancer and screening uptake

**Fig. 6:**
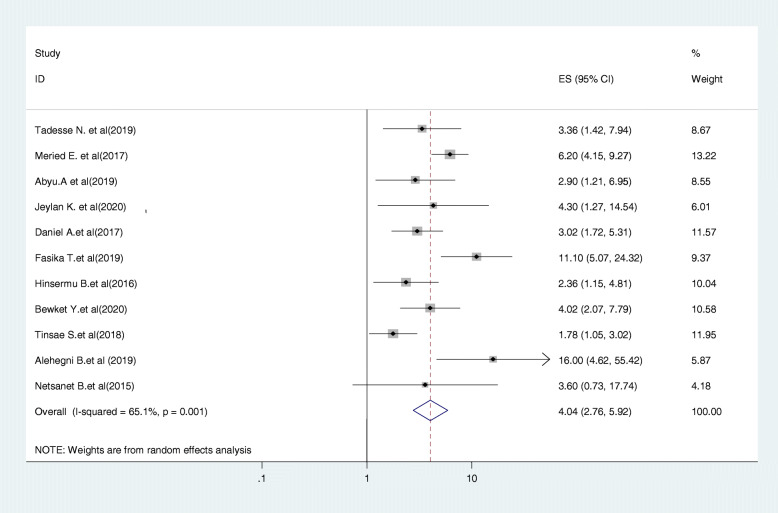
Forest plot displaying the association between knowledge of cervical cancer and screening and screening uptake in Ethiopia

Eleven studies were included in this category of meta-analysis [22, 23, 25–30, 32, 33, 37]. Women who had adequate knowledge about cervical cancer screening were 4.04 times (OR = 4.04, 95% CI:2.76, 5.92) more likely to be screened as compared to those who had no adequate knowledge about cervical cancer screening. In this meta-analysis, included studies were characterized by the existence of a moderate heterogeneity (I^2^ = 65.1%, *P* = 0.026). Moreover, we used a random-effect model analysis (Fig. 6).

#### Relationship between history of multiple sexual partners and cervical cancer screening

**Fig. 7:**
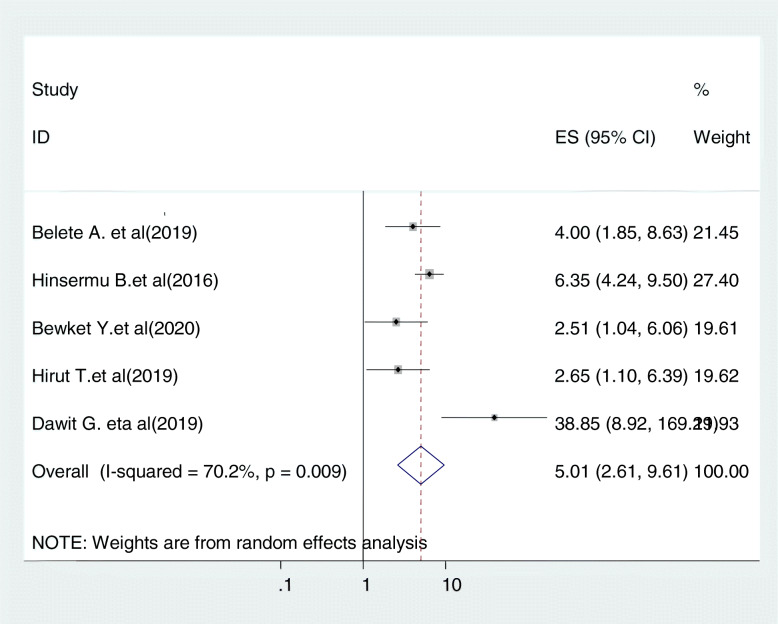
Relationship between history of multiple sexual partners and cervical cancer screening

Five studies were included in this category of meta-analysis [24, 30, 31, 41, 42]. The likelihood of screening for cervical cancer among women with history of multiple sexual partners were 5.01 times (OR = 5.01, 95% CI: 2.61, 9.61) more likely to be screened for cervical cancer as compared to their counter parts. In this meta-analysis, included studies were characterized by existence moderate heterogeneity (I^2^ = 70.2%, *P* = 0.009). As a result, we used a random-effect model analysis (Fig. 7).

#### Relationship between women’s age and cervical cancer screening

**Fig. 8:**
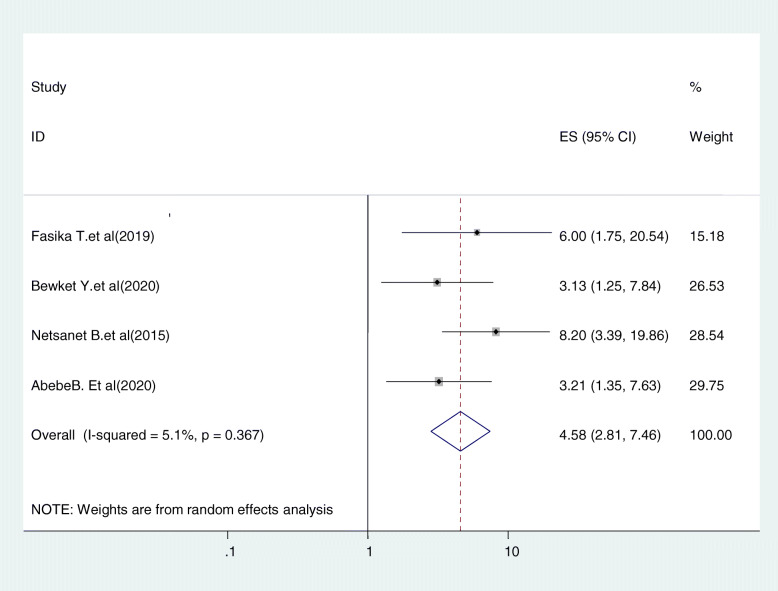
Relationship between women’s age and cervical cancer screening

Five studies were included in this category of meta-analysis [27, 31, 35, 37]. Women who were in their 30’s were 4.58 times (OR = 4.58, 95%CI:2.81,7.46) more likely to uptake cervical cancer screening service as compared to those who were in the age range of 21–29. In this meta-analysis, included studies were characterized by existence of low heterogeneity (I^2^ = 5.0%, *P* = 0.367). Moreover, we used a random-effect model analysis (Fig. 8).

#### Relationship between history of sexually transmitted disease and cervical cancer screening uptake

**Fig. 9:**
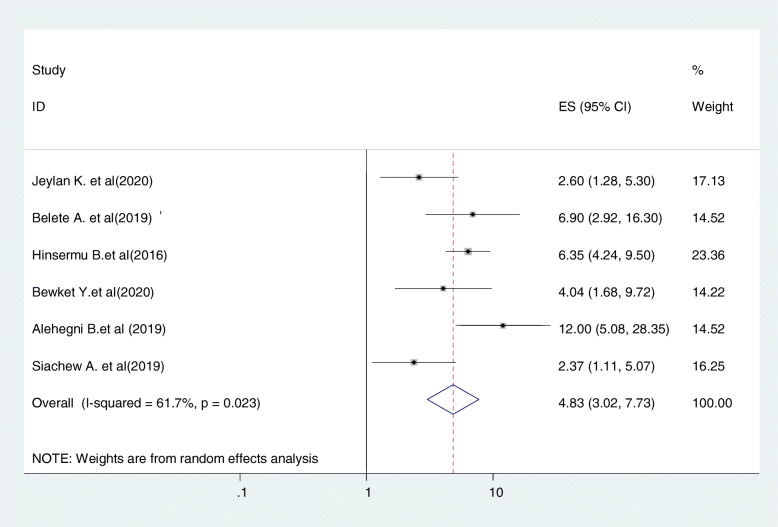
Relationship between history of sexually transmitted disease and cervical cancer screening uptake

Six studies were included in this category of meta-analysis [23, 24, 30, 31, 33, 34]. Women who have admitted having history of multiple sexual partners were 4.8 times (OR = 4.8, 95%CI:3.8, 7.7) more likely to undergo screening for cervical cancer as compared to those who did not have such history. In this meta-analysis, included studies were characterized by moderate heterogeneity (I^2^ = 61.7%, *P* = 0.023). Moreover, we used a random-effect model analysis (Fig. 9).

#### Relationship between perceived susceptibility to cervical cancer and cervical cancer screening uptake

**Fig. 10:**
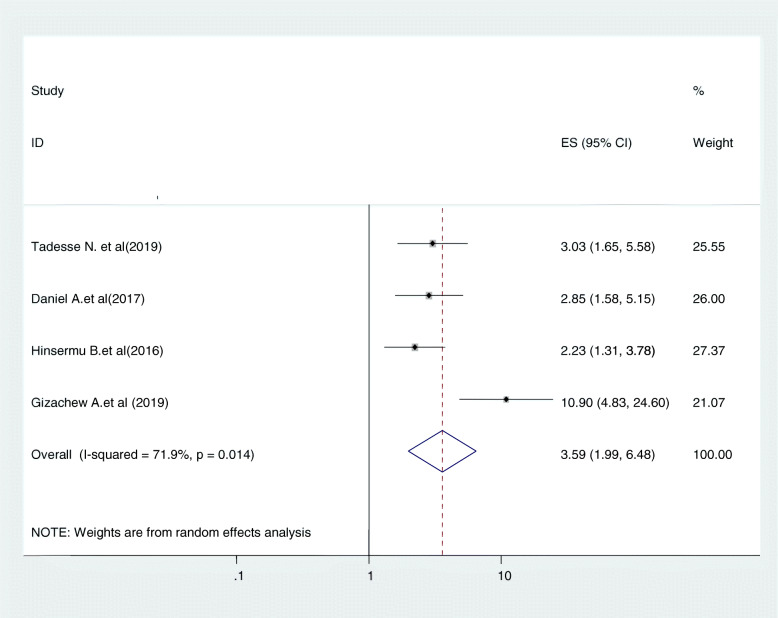
relationship between perceived susceptibility to cervical cancer and cervical cancer screening uptake

Women’s perception about potential susceptibility to cervical cancer was another determinant factor for cervical cancer screening uptake. Four studies were included in this category of meta-analysis [25, 29, 30, 43]. The likelihood of cervical cancer screening uptake among women who had perceived susceptibility to cervical cancer were nearly 3.6 times (OR = 3.59, 95% CI:1.99,6.49) more likely to be screened for cancer of cervix than their counter parts. In this meta-analysis, included studies were characterized by moderate heterogeneity (I^2^ = 71.9%, *P* = 0.014). Furthermore, we computed a random effect meta-analysis (Fig. 10).

#### Relationship between getting advice from health care providers and cervical cancer screening uptake

**Fig. 11:**
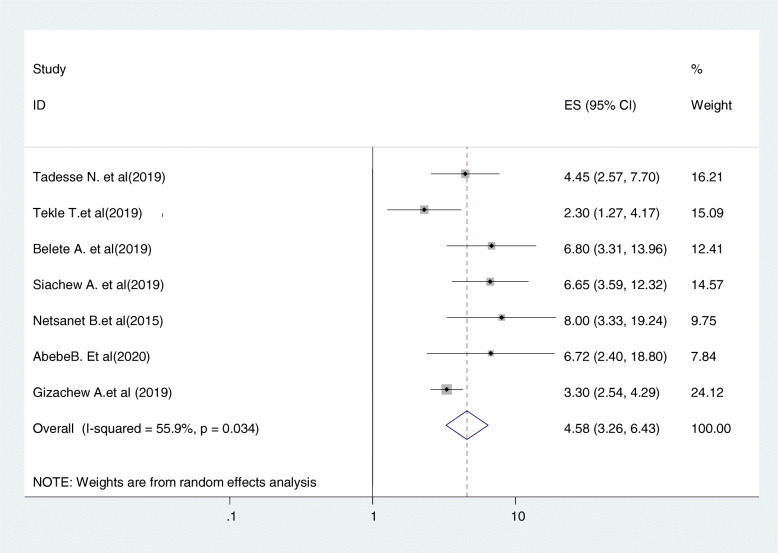
relationship between getting advice from health care providers and cervical cancer screening uptake

Seven studies were included in this category of meta-analysis [21, 24, 29, 34, 35, 37, 43]. The likelihood of up taking cervical cancer screening among women who get advice from health care providers were nearly 4.6 times (OR = 4.58, 95% CI:3.26,6.43) more likely to be screened for cancer of cervix as compared to women who did not get medical advice from health care providers. In this meta-analysis, included studies were characterized by the existence of moderate heterogeneity (I^2^ = 55.9%, *P* = 0.034). Furthermore, we computed a fixed effect meta-analysis (Fig. 11).

#### Relationship between women’s educational level and cervical cancer screening uptake

**Fig. 12:**
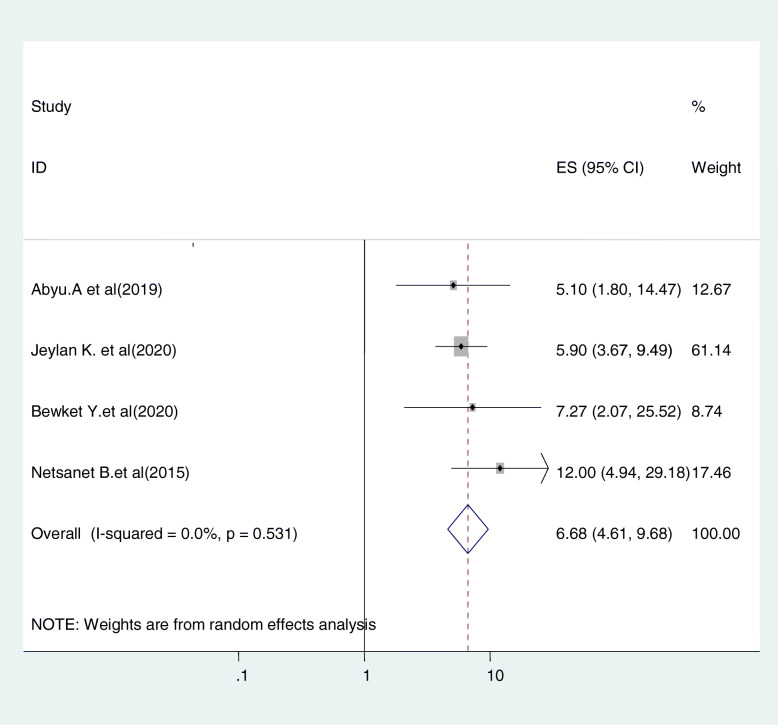
relationship between women’s educational level and cervical cancer screening uptake

Four studies were included in this category of meta-analysis [22, 23, 31, 37]. Women who finished primary education and above were 6.68 times (OR = 6.68, 95%CI: 4.61, 9.68) more likely to be screened for cervical cancer as compared to uneducated women. In this meta-analysis, included studies were characterized by the existence of no heterogeneity (I^2^ = o.o%, *P* = 0.531). Moreover, we used a fixed-effect model analysis (Fig. 12).

#### Relationship between women’s attitude and cervical cancer screening uptake

**Fig. 13:**
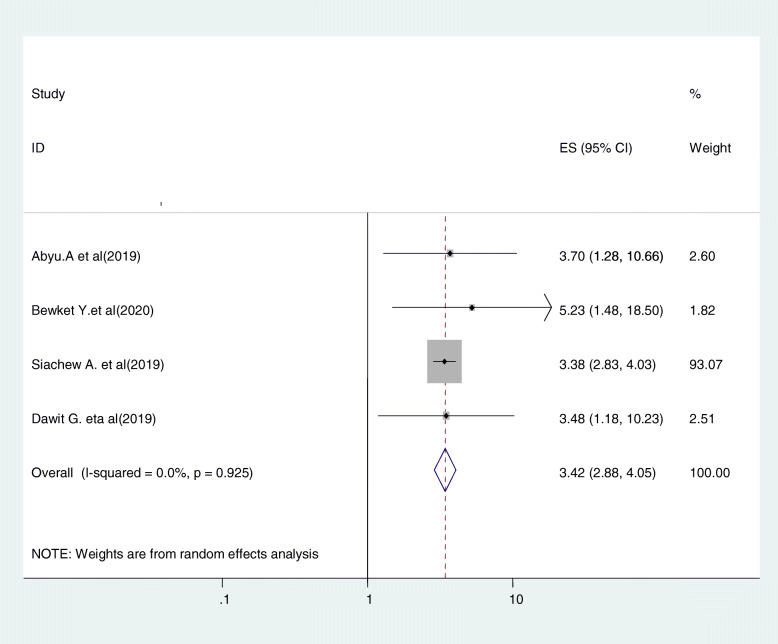
Relationship between women’s attitude and cervical cancer screening uptake

Five studies were included in this category of meta-analysis [22, 31, 34, 42]. The likelihood of screening for cervical cancer among women with favorable attitude towards cervical cancer and screening were 3.42 times (OR = 3.42, 95% CI: 2.88, 4.05) more likely to be screened for cervical cancer than women who had unfavorable attitude. In this meta-analysis, included studies were characterized by no existence of heterogeneity (I^2^ = 0.0%, *P* = 0.925). Moreover, we used a fixed-effect model analysis due to the absence of heterogeneity (Fig. 13).

## Discussion

Early treatment and routine cervical cancer screening can prevent up to 80% of cervical cancers, if cervical abnormalities are identified at stages when they can be easily treated. To identify precancerous lesions, WHO recommends screening for all women aged 30 to − 49 years, which are usually asymptomatic. HPV vaccination is vital to prevent cervical cancer but does not replace the necessity of cervical cancer screening and early treatment in women [45].

In this review, 24 [24] studies comprising a total of 14,582 participants were analyzed to estimate the best available evidence for the prevalence and factors associated with cervical cancer screening among age eligible women in Ethiopia. Accordingly, the pooled prevalence of cervical cancer screening was 13.46% (95%CI: 11.06, 15.86). The result is lower than the study findings in Canada 58% [46], England 85.7% [47], Catalonia 50.6% [48], and Kenya 46% [49]. The possible reason for this variation could be due to differences in socio-demographic and economic status of the study respondents as well as the countries’ health policy variations like institutional framework to promote screening, which could have largely succeeded in implementing successful programs regarding cervical cancer screening.

Another possible reason for may be due to uneven distribution of screening services centers. For example; there is universal access to health care in Canada, including the availability of primary care and specialist physicians, which differs from other health care models [46]. Moreover, Kenya has a more robust cervical cancer screening program; as a result, there is increased awareness about cervical cancer and its screening [50].

The finding of this systematic review is higher than the study conducted in Ghana (2.4%) [51]. The possible reason for the low coverage of cervical cancer screening services in Ghana might be there is still no national policy or program regarding cervical cancer screening and that could be contributing to the low screening of cervical cancer in Ghana. The other possible reason could be the ignorance about the disease and its screening practices as well as perceptions and attitudes based on cultural and religious beliefs.

This research revealed women’s knowledge on cervical cancer and screening is an implication in screening uptake. Women who were knowledgeable on cervical cancer and its screening were about 4 times more likely to uptake screening services than women who were not knowledgeable (AOR =4.04, 95% CI:2.76, 5.92). The result is supported by studies done in Tanzania [52], Botswana [53], and China [54]. The possible reason might be explained by the fact that the increasing of women’s knowledge about cervical cancer and the benefits of screening directly lead women to utilize the screening service.

History of multiple sexual partners is also an important predictor of cervical cancer screening uptake. Woman who had two or more life time sexual partners were 5 times (OR = 5.01, 95% CI: 2.6,9.61) more likely to undergo screening for cervical cancer than those who had less than two life time sexual partner. The finding is consistent with study conducted in Nigeria [55], Africa [56], and Botswana [53]. The possible explanation could be the more sexual partners a woman has, the greater are her chances of becoming infected with human immune deficient virus and other sexually transmitted disease including human Papilloma virus, the most common risk factors for development of cervical cancer. Therefore, they would have the chance to be infected with sexually transmitted disease with its signs and symptoms which increases health facility visits. Moreover, they might get more healthcare counseling about human papiloma virus, precervical cancer, invasive cervical cancer, and screening that promote screening service uptake.

Women’s age is the other predictor of cervical cancer screening uptake. Women in their 30’s were 4.58 times more likely to be screened compared to women in their 20s (OR = 4.58, 95%CI:2.81,7.46). The lower screening rates among younger (21–29 years) women is not unique to Ethiopia; there are also researches with the same findings from elsewhere in developed countries [57] and Australia [56]. The explanation for this could be that the bimodal distribution of cervical cancer, one at 30s and other at 60s. These two age groups are the ages at which cervical lesions become symptomatic. As a result, women see themselves as being at risk of invasive cervical cancer and seek medical care and screening services. Additionally, in Ethiopia, the cervical cancer screening guideline promotes women age of 30–49 to be screened for cervical cancer and women of this age group might have better knowledge and intention to be screened. Moreover, this age group is a productive age and a chance of getting more gynecological examination, giving birth, and getting more health information about sexual and reproductive health including cervical cancer that promote screening service.

Women who have been diagnosed for sexually transmitted disease were nearly 5 times (OR = 4.83, 95%CI: 3.02,7.73) more likely to be screened than those who have no history of sexually transmitted diseases. The result is in line with study result in Botswana [53], and Zambia [58]. The above association could be explained by being infected by sexually transmitted diseases like HIV, HPV, and others with symptoms to promote the chance of seeking medical help, gynecological examination, and medical information about the deadly cervical cancer and screening that in turn promote screening.

The findings of our study revealed that women’s educational level has a positive effect on cervical cancer screening uptake. Women who had primary and level of education were nearly 7 times (OR = 6.68, 95%CI: 4.61, 9.68) more likely to undergo screened for cervical cancer than those with no formal educational levels. The same finding was observed in studies done in India [59], Nigeria [60], Ghana [61], and Italy [62] . This is not surprising as we expect those women who are more educated to have an understanding of the cause, risk factors, prevention mechanism and treatment of cervical cancer and as such can demand screening services. Furthermore, better-educated women have a higher efficiency in understanding health education as well as impart self-efficacy, confidence, and motivation, in search for health interventions for their health including cervical cancer.

Women’s perception about potential susceptibility to cervical cancer was another determinant factor for cervical cancer screening uptake. Women who have receptive perception about potential susceptibility to develop cervical cancer in the future were 3.6 times (OR = 3.59, 95%CI:1.99,6.48) more likely to undergo screening than those who have non-receptive perception. This result is similar to the findings of a study done in Uganda [63]. This could be explained by women’s view of own vulnerability to illness, and if they perceived that they are prone to cancer of cervix, they seek screening and medical care to protect themselves.

Women who had advice/consultation from health care providers were 4.58 more (OR = 3.26, 6.43) likely to be screened when compared with women who had no advice. This finding is consistent with a study conducted in Jamaica [64], and Tanzania [65]. This may be due to the information from health care providers, to increase awareness about cervical cancer, and the advantages of having screening services to prevent deadly invasive cervical cancer.

Women’s attitude towards cervical cancer and its screening had been associated with cervical cancer screening uptake. Women who had favorable attitude towards cervical cancer and screening were 3.42 times (OR = 3.4, 95%CI: 2.88, 4.05) more likely to undergo screening than those who have unfavorable attitude. This finding is shared with a previous study conducted in Nigeria [66], Ghana [67], and Thailand [68]. The reason might be having a favorable attitude is mostly followed by having an understanding of the cancer of cervix, the benefit of screening, and engagement in cervical screening as well.

### Limitations

This meta-analysis was included only articles conducted in the English language, which might have been restricted, some papers being included. All the included articles were cross-sectional; as a result, the outcome variables might be affected by other confounding variables in nature and the temporal cause-and-effect relationship may not be well addressed via cross-sectional studies.

## Conclusions

Cervical cancer is a preventable disease. Knowledge of the disease, early screening and treatment could decrease the mortality associated with it. In Ethiopia, most women seek medical help after reached an advanced form of the disease due to lack of awareness and community level interventions to encourage screening. The overall prevalence of cervical cancer screening is still remarkably low. Women’s knowledge about cervical cancer and screening, history of multiple sexual partners, women’s age, history of sexually transmitted disease, perceived susceptibility to cervical cancer, getting advice from health care providers, women’s educational level, women’s attitude towards cervical cancer and its screening were the determinant factors of cervical cancer screening uptake in Ethiopia. Therefore, to increase the uptake of cervical cancer screening among age-eligible women, it is better to create awareness programs early detection and treatment of cervical cancer, and promotion through the mass media, and health talks about cervical cancer screening, and the available facilities. Opportunistic screening in health facilities could be promoted to improve cervical cancer screening uptake, for all age-eligible women. Moreover, to promote cervical cancer screening, it is better to integrate cancer control programmers’ into existing primary sexual and reproductive health care services, strengthen multi-sectoral collaboration, and improve public health awareness to tackle the devastating effect of cervical cancer. It is also important to inform that every woman is susceptible to cervical cancer, especially after starting sexual intercourse, and screening remains fundamental in the fight against cervical cancer before becoming invasive or deadly. Health literacy that teaches the step-by-step practice of cervical screening to promote favorable attitudes towards screening and to improve the confidence of women to undergo screening is also recommended.

## Data Availability

All datasets have been presented within the manuscript. The datasets supporting the conclusions of this article is available from the corresponding authors on request.
